# The genome sequence of Artibeus intermedius (Chiroptera, Phyllostomidae, Stenodermatinae; J. A. Allen, 1897)

**DOI:** 10.12688/wellcomeopenres.23865.1

**Published:** 2025-06-09

**Authors:** Nancy B. Simmons, Melissa R. Ingala, Brian P. O'Toole, Kirsty McCaffrey, Giulio Formenti, Philip Philge, Shipra Gupta, Erich D. Jarvis, Ning Zhang, Jonathan L. Gray, Nadolina Brajuka, Myrtani Pieri, Meike Mai, Emma C. Teeling, Sonja C. Vernes, The Bat Biology Foundation, The Bat1K Consortium

**Affiliations:** 1 Department of Mammalogy, Division of Vertebrate Zoology, American Museum of Natural History, New York, NY 10024, USA; 2 Department of Biological Sciences, Fairleigh Dickinson University, Madison, New Jersey, NJ 07940, USA; 3 Division of Mammals, Department of Vertebrate Zoology, National Museum of Natural History, Smithsonian Institution, Washington, District of Columbia, DC 20560, USA; 4 Paratus Sciences, New York, USA; 5 Vertebrate Genome Lab, The Rockefeller University, New York NY, USA; 6 Excelra, Hyderabad, India; 7 Department of Life Sciences, School of Life and Health Sciences, University of Nicosia, Nicosia, 2417, Cyprus; 8 School of Biology, University of St Andrews, St Andrews, UK; 9 School of Biology and Environmental Science, University College Dublin, Dublin, Leinster, Ireland; 10 Wellcome Sanger Institute, Wellcome Genome Campus, Cambridgeshire, CB10 1SA, UK

**Keywords:** Artibeus intermedius, genome sequence, chromosomal, Bat1K

## Abstract

We present a genome assembly from an individual male *Artibeus intermedius* (Chordata; Mammalia; Chiroptera; Phyllostomidae). The genome sequence is 2.3Gb in span. The majority of the assembly is scaffolded into 17 chromosomal pseudomolecules, with the X and Y1/Y2 sex chromosomes assembled.

## Species taxonomy

Eukaryota; Metazoa; Chordata; Craniata; Vertebrata; Euteleostomi; Mammalia; Eutheria; Laurasiatheria; Chiroptera; Yangochiroptera; Phyllostomidae; Stenodermatinae; Stenodermatini; Artibeina; *Artibeus*; *Artibeus intermedius* ( Baker *et al*., 2016; Cirranello *et al*., 2016; Simmons & Cirranello, 2025).

## Introduction

**Figure 1.**
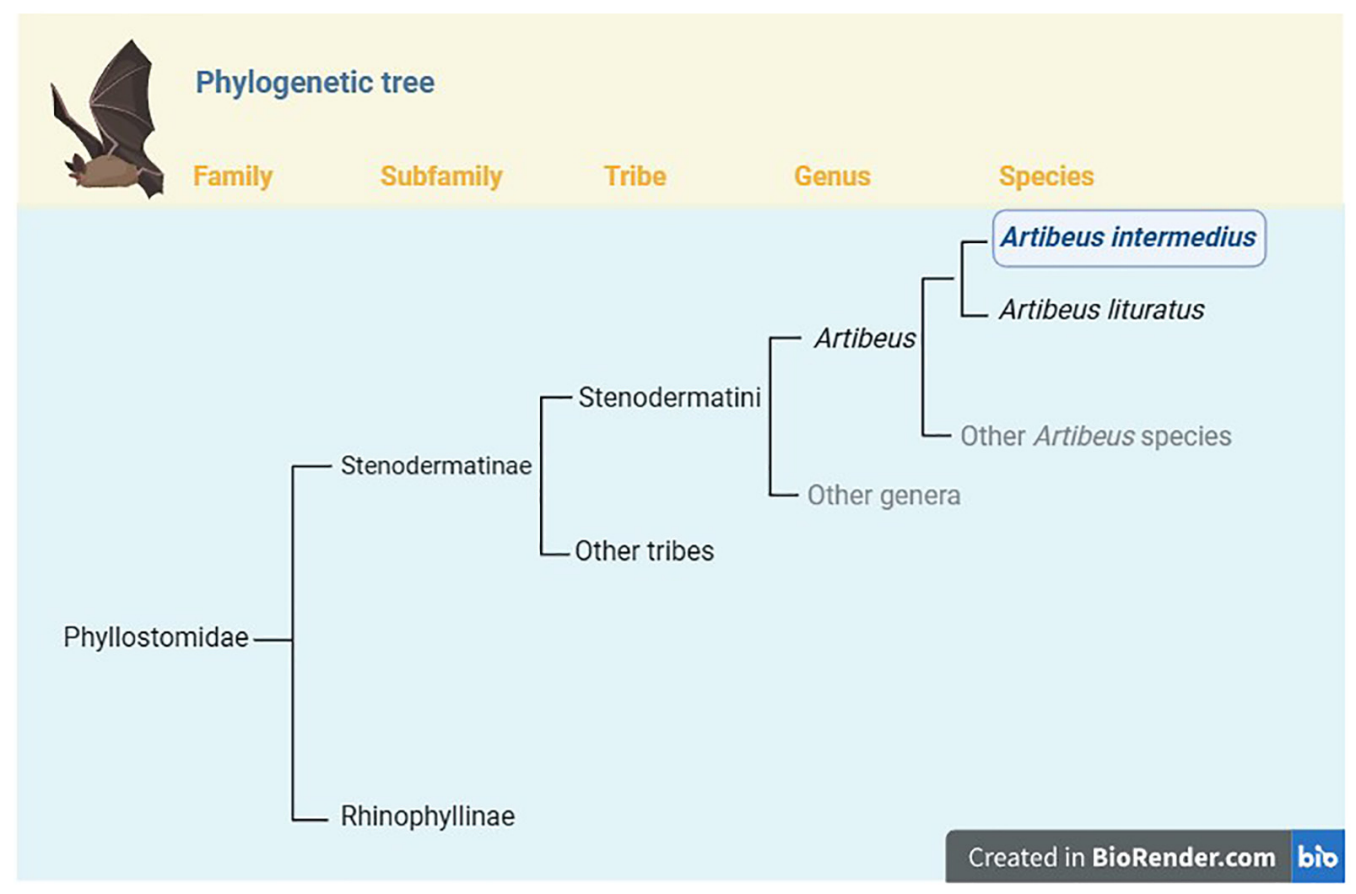
Position of *Artibeus intermedius* in the phylogeny of Family Phyllostomidae. *Artibeus intermedius* is one of 13 species currently recognized in the genus *Artibeus* ( Simmons & Cirranello, 2025). *Artibeus* belongs to the Tribe Stenodermatini in the Subfamily Stenodermatinae, which currently includes 19 genera and 77 species ( Baker *et al*., 2016; Cirranello *et al*., 2016; Simmons & Cirranello, 2025). Within *Artibeus*, the closest relative of *A. intermedius* is *A. literatus*; until recently these taxa were considered conspecific. Phylogeny based on Botero-Castro *et al.* (2013), Larsen *et al.* (2013), and Rojas *et al.* (2016).

The genus *Artibeus* is a clade of relatively large-bodied frugivorous bats belonging to the Tribe Stenodermatini within the Subfamily Stenodermatinae ( Figure 1; Baker *et al*., 2016; Cirranello *et al*., 2016; Simmons & Cirranello, 2025). *Artibeus* are generally believed to be figure specialists although they also consume a wide variety of other fruits ( de Souza Laurindo & Vinzentin-Bugoni, 2020; Emmons & Feer, 1997; Reid, 2009). *Artibeus* bats are common throughout the Neotropics with multiple species often occurring in sympatry ( Emmons & Feer, 1997; Larsen *et al*., 2013; Marques-Aguiar, 1994; Marques-Aguiar, 2007; Reid, 2009). *Artibeus intermedius* J. A. Allen, 1897, which is among the larger members of this group, occurs in Mexico, Belize, Guatemala, Honduras, El Salvador, Nicaragua, Costa Rica, and Panama in lowland areas up to about 1700 m ( Larsen *et al*., 2013; Mammal Diversity Database, 2024; Reid, 2009). Although some sources indicate that the range of *A. intermedius* may extend into Colombia in northern South America (e.g., Koopman, 1994; Reid, 2009) this has not been confirmed, and recent studies suggest that the southern limit of the range is Pamana (e.g., Larsen *et al*., 2013). 

The taxonomic status of *Artibeus intermedius* has been the source of debate. Some authors have suggested that *A. intermedius* should be considered a junior synonym of *A. lituratus* (e.g., Guerrero *et al*., 2008; Hoofer *et al*., 2008; Lim *et al*., 2004; Marques-Aguiar, 1994; Redondo *et al*., 2008; Simmons, 2005) but others have concluded that these are different species (e.g., Davis, 1984; Koopman, 1994; Larsen *et al*., 2013; Marchán-Rivadeneira *et al*., 2012; Reid, 2009; Wilson, 1991). Recent comprehensive bat classifications (e.g., Mammal Diversity Database, 2024; Simmons & Cirranello, 2025) follow Larsen *et al*. (2013) in treating *A intermedius* as a species distinct from *A. lituratus*. Both species apparently occur in sympatry at the site in Belize where the individual reported below was captured (see Methods). The published genome sequence may be useful for resolving this taxonomic debate.

**Figure 2.**
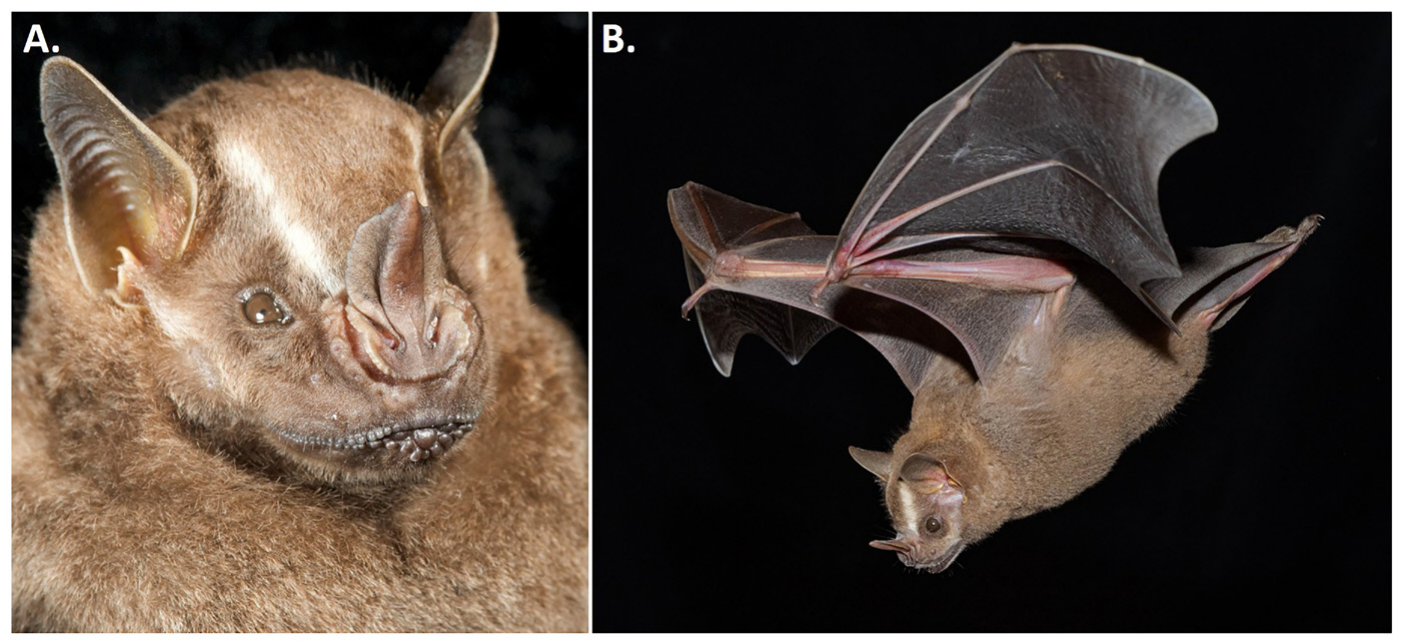
*Artibeus intermedius.* Adult individuals of *Artibeus intermedius*. **A**, portrait showing facial striping, noseleaf, and chin morphology; and **B**, in flight. [Photos taken at Lamanai, Belize by Brock and Sherri Fenton ( **A**) and Price Sewall ( **B**)].

*Artibeus intermedius* ( Figure 2) can be distinguished from congeners based on a series of morphological traits including the following: large size (forearm 61–69 mm, greatest length of skull 28–31 mm, 40–54 g), prominent and well-defined white facial stripes with lateral stripe less well developed than medial stripe, brown or golden brown dorsal fur and brown wings, ventral fur that is not conspicuously frosted with white, uropatagium and legs that are well furred, preorbital and postorbital processes well developed, and M3 absent but m3 present ( Koopman, 1994; Reid, 2009).

*Artibeus intermedius* is common and abundant in many types of forest including lowland primary and secondary forest, pine-oak and evergreen highland forest, and agricultural fruit groves ( Reid, 2009). Like other *Artibeus* species, they are thought to be fig specialists, although they also consume a wide range of other fruit including *Cercropia*, *Solanum*, *Manilkara*, and *Stenocerus*, depending on the habitat ( García-Estrada *et al*., 2012; Herrera *et al*., 2017; Ingala *et al*., 2021). They also occasionally consume insects including *Hymenoptera, Diptera, Hemiptera*, and *Coleoptera* ( Herrera *et al*., 2017; Ingala *et al*. 2021). Davis (1984) reported that *Artibeus intermedius* roosts alone or in small groups near the mouth of caves or in shaded crevices, but all other records involve use of foliage or trees. Reid (2009) reported that *A. intermedius* roosts under foliage and may occupy leaf tents in tall, palmate palm trees. Using radiotelemetry, Evelyn and Stiles (2003) found that these bats roosted externally on branches and vines and under palm leaves, and that they select trees of small diameter that are close to neighboring taller trees, likely providing cryptic roosts under multiple overlapping crowns. Males generally roost alone, while females roost in small groups ( Evelyn & Stiles, 2003).

*Artibeus intermedius* has not been assessed by the IUCN Redlist of Threatened Species because it was treated as conspecific with *A. literatus* following Simmons (2005) in prior assessments of Neotropical bats. However, because of its wide distribution, use of many habitat types, presumed large population size, and lack of any major threats, it seems unlikely that it will be classified as anything other than Least Concern when it is assessed in the future.

*Artibeus intermedius* has been used in experimental studies of viral pathogens including rabies and dengue virus, and these bats are known to host a variety of bacterial pathogens ( Becker *et al*., 2020; Obregón-Morales *et al*., 2017; Perea-Martínez *et al*., 2013; Pierlé *et al*., 2015).

## Genome sequence report

The genome was sequenced from a single male *Artibeus intermedius* (field number BZ-126, catalog number AMNH:Mammalogy:280690) collected at the Lamanai Archaeological Reserve, Orange Walk District, Belize, on 9 November 2021. A total of 40x-fold coverage in Pacific Biosciences Hi-Fi long reads was generated after removal of all reads shorter than 10kb. HiFiasm in Hi-C phasing mode was used to generate contigs of each haplotype 1 (mArtInt1.hap1) and haplotype 2 (mArtInt1.hap1). The contigs of each haplotype were scaffolded with the same Hi-C data. The assembly was curated for structural errors and to name chromosomes. Haplotype 1 was the more contiguous assembly with higher quality metrics, and selected as the reference, with both sex chromosomes moved into it. This final assembly has a total length of 2.3 Gb in 419 sequence scaffolds with a scaffold N50 of 160.4 Mb. The majority, 98.5%, of the assembly sequence was assigned to 17 chromosomal-level scaffolds, representing 14 autosomes (numbered by sequence length), and the X chromosome and two Y (Y1/Y2) sex chromosomes ( Table 1). The assembly has a BUSCO ( Simao *et al.*, 2015) completeness of 96.5% using the laurasiatheria reference set.

**Table 1.** Chromosomal pseudomolecules in the genome assembly of *Artibeus intermedius*. ENA accession Chromosome Size (Mb) GC%. The chromosome number of *Artibeus intermedius* is 2n=[15].

ENA accession	Chromosome	Size (Mb)	GC%
SUPER_1	1	245407277	0.4049
SUPER_2	2	215736802	0.4017
SUPER_3	3	188874488	0.4201
SUPER_4	4	182636357	0.4269
SUPER_5	5	176201619	0.4179
SUPER_6	6	160430197	0.4333
SUPER_7	7	151570710	0.4161
SUPER_8	8	149657484	0.4128
SUPER_9	9	144924135	0.4102
SUPER_10	10	133091569	0.4372
SUPER_11	11	119577359	0.4496
SUPER_X	X	110131259	0.4275
SUPER_12	12	101253383	0.3993
SUPER_Y1	Y1	61536898	0.4643
SUPER_13	13	58707028	0.4534
SUPER_14	14	37838280	0.4475
SUPER_Y2	Y2	22583885	0.4309

## Methods

The *Artibeus intermedius* specimen was a male individual collected on an American Museum of Natural History (AMNH) field expedition at the Lamanai Archaeological Reserve in the Orange Walk District of Belize. The individual sampled was identified as *A. intermedius* based on morphometrics (e.g., forearm length, body mass) and morphological traits (e.g., brightness of eye stripes, fur color and distribution) as described above. The bat was caught in a ground-level mist net set near the Stela Temple in the Lamanai Archaeological Reserve (17.76639 N, 88.65225 W). All efforts were made to minimize any distress or suffering by the animal. The individual sampled was subjected to minimal handling after capture, and it was held in a clean cloth bag after capture as per best practices for field containment of bats. After species identification, the individual was euthanized humanely the same night it was captured. The animal was euthanized by isoflurane inhalation (<1 ml to moisten cotton ball, formula CHF _2_OCClHCF _3_, CAS number 26675-46-7; Manufacturer Piramal Critical Care, Supplier US Pharmacy Systems, Product code 5034-1FL-SOL-ORA), an approved and humane euthanasia method that rapidly causes unconsciousness within seconds and death within a minute or two with no significant suffering by the animal. Capture and sampling were conducted under Belize Forest Department Permit FD/WL/1/21(16) and Belize Institute of Archaeology Permit IA/S/5/6/21(01), and samples were exported under Belize Forest Department permit FD/WL/7/22(08). All work was conducted with approval by the AMNH Institutional Animal Care and Use Committee (AMNHIACUC-20191212). All data were recorded and reported in accordance with the ARRIVE guidelines ( Kilkenny *et al.*, 2010) – see data availability section and Table 2. Tissues were removed from the subject individual immediately following euthanasia and were flash-frozen in a liquid nitrogen dry shipper, with the cold chain maintained from field to museum to laboratory.

DNA was extracted using Nanobind extraction from muscle tissue following the Circulomics Nanobind HMW DNA Extraction Protocol. Pacific Biosciences HiFi libraries were constructed according to the manufacturer's instructions. Hi-C data was generated using the Arima Hi-C+ High Coverage kit from liver tissue sample. Sequencing was performed by the Genomic Operations DNA Pipelines at Paratus Sciences on Pacific Biosciences Sequel IIe (HiFi reads) and Illumina NextSeq 2000 (Hi-C) instruments.

**Table 2.** Genome data for *Artibeus intermedius*.

*Project accession data*	
Assembly identifier	GCA_038363225.1
Species	*Artibeus intermedius*
Specimen	mArtInt1
NCBI taxonomy ID	51014
BioProject	Bat1K: Accession: PRJNA489245; ID: 489245
BioSample ID	SAMN40002248
Isolate information	Male - Muscle & Liver
Assembly accession haplotype 1 and main reference	GCA_038363145.1
Assembly accession haplotype 2	GCA_038363225.1
Haplotype 1 Span (Mb)	2.3Gb
Number of contigs hap1	416
Contig N50 length (Mb) hap1	60
Number of scaffolds	443
Scaffold N50 length (Mb)	160.4
Longest scaffold (Mb)	245

* BUSCO scores based on the mammalia_odb10 BUSCO set using v5.0.0. C= complete [S= single copy, D=duplicated], F=fragmented, M=missing, n=number of orthologues in comparison. * *Artibeus intermedius* BUSCO scores based on laurasiatheria_odb10 BUSCO set v5.3.2.

**Figure 3.**
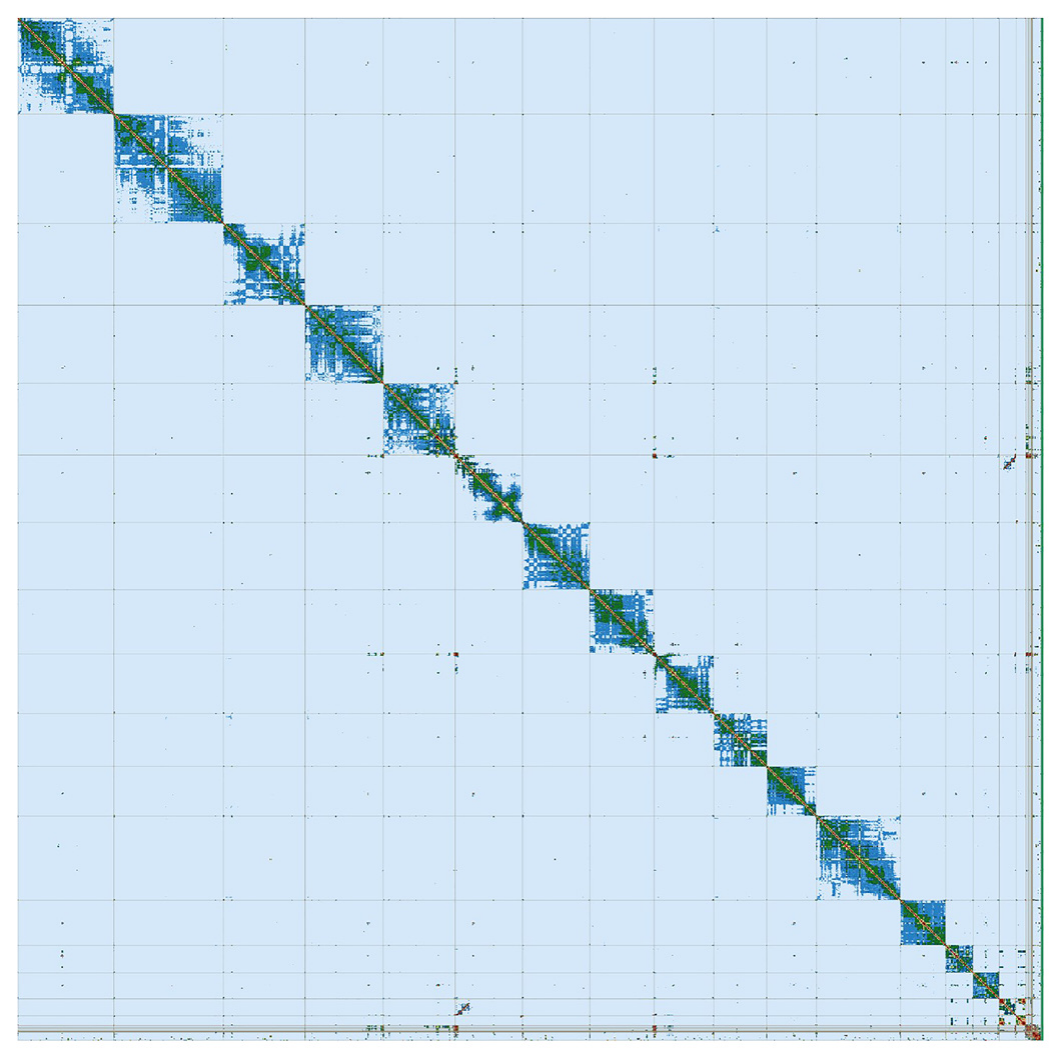
Hi-C Contact Map of the *Artibeus intermedius* assembly with 14 chromosomes and 3 sex chromosomes, visualized using PretextView. Hi-C Contact Map of the *Artibeus intermedius* assembly done in collaboration with the VGL.

**Figure 4.**
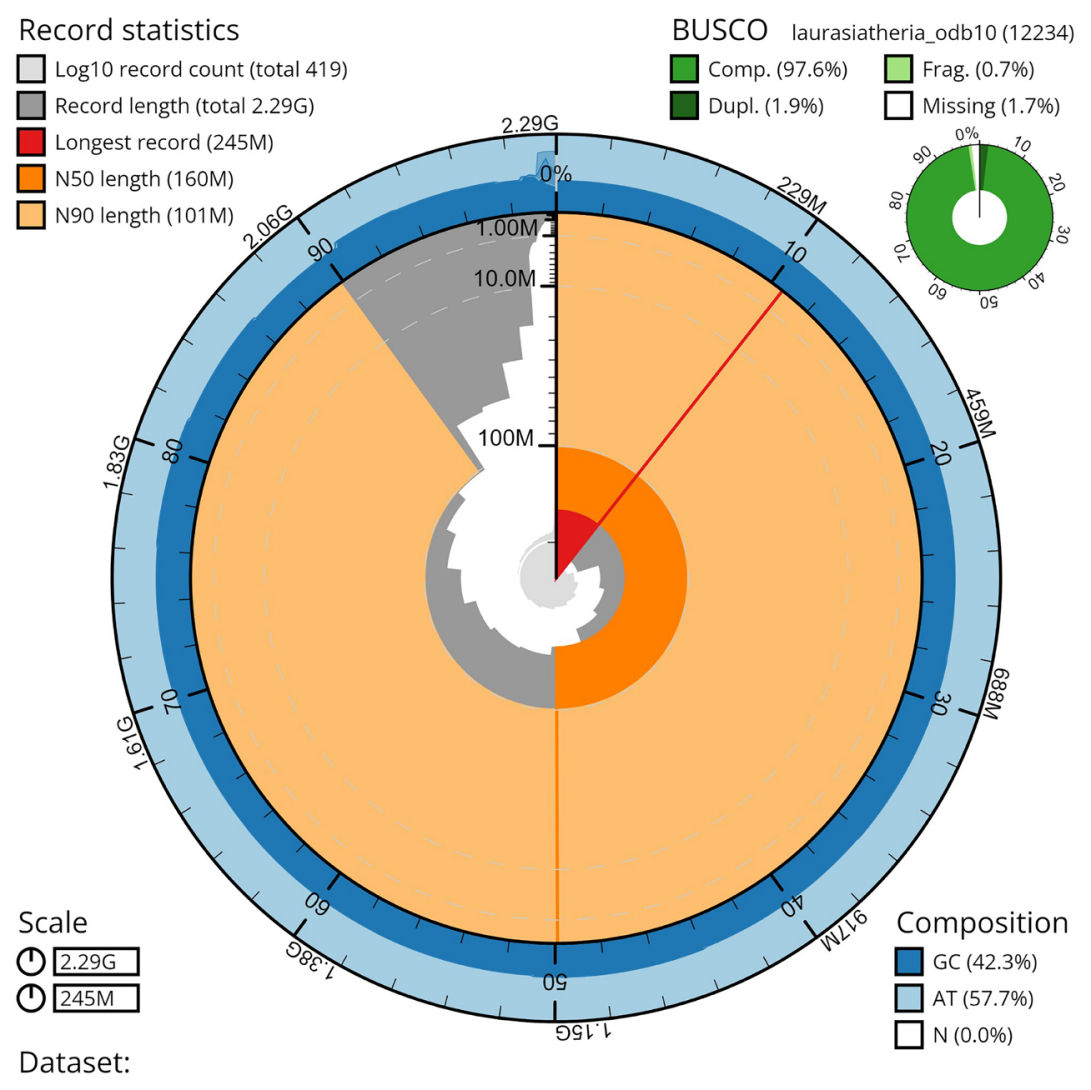
Genome assembly metrics generated using blobtoolkit for the *Artibeus intermedius* genome assembly. The larger snail plot depicts scaffold statistics including N50 length (bright orange) and base composition (blue). The smaller plot shows BUSCO completeness in green. Done in collaboration with Excelra through Paratus Sciences.

Assembly was carried out following the Vertebrate Genome Project Galaxy pipeline v2.0 ( Lariviere *et al*., 2024). A brief synopsis of the method is as follows: Genome size was estimated using GenomeScope2 ( Vurture *et al*., 2017). Hifiasm with Hi-C phasing was used for assembly of each haplotype ( Cheng *et al*., 2021). Scaffolding of the contigs or each haplotype with Hi-C data ( Rao *et al*., 2014) was carried out with YaHS ( Zhou *et al*., 2023). PretextView was implemented to generate a Hi-C contact map used for manual curation, and errors fixed and chromosomes named (haplotype 1 in Figure 3). The quality of the assemblies were evaluated using Merqury ( Nurk *et al*., 2020) and BUSCO ( Manni *et al*., 2021). Figure 4 – Figure 6 were generated using BlobToolKit ( Challis *et al*., 2020). Software utilised for the *A. intermedius* analysis are depicted in Table 3.

**Figure 5.**
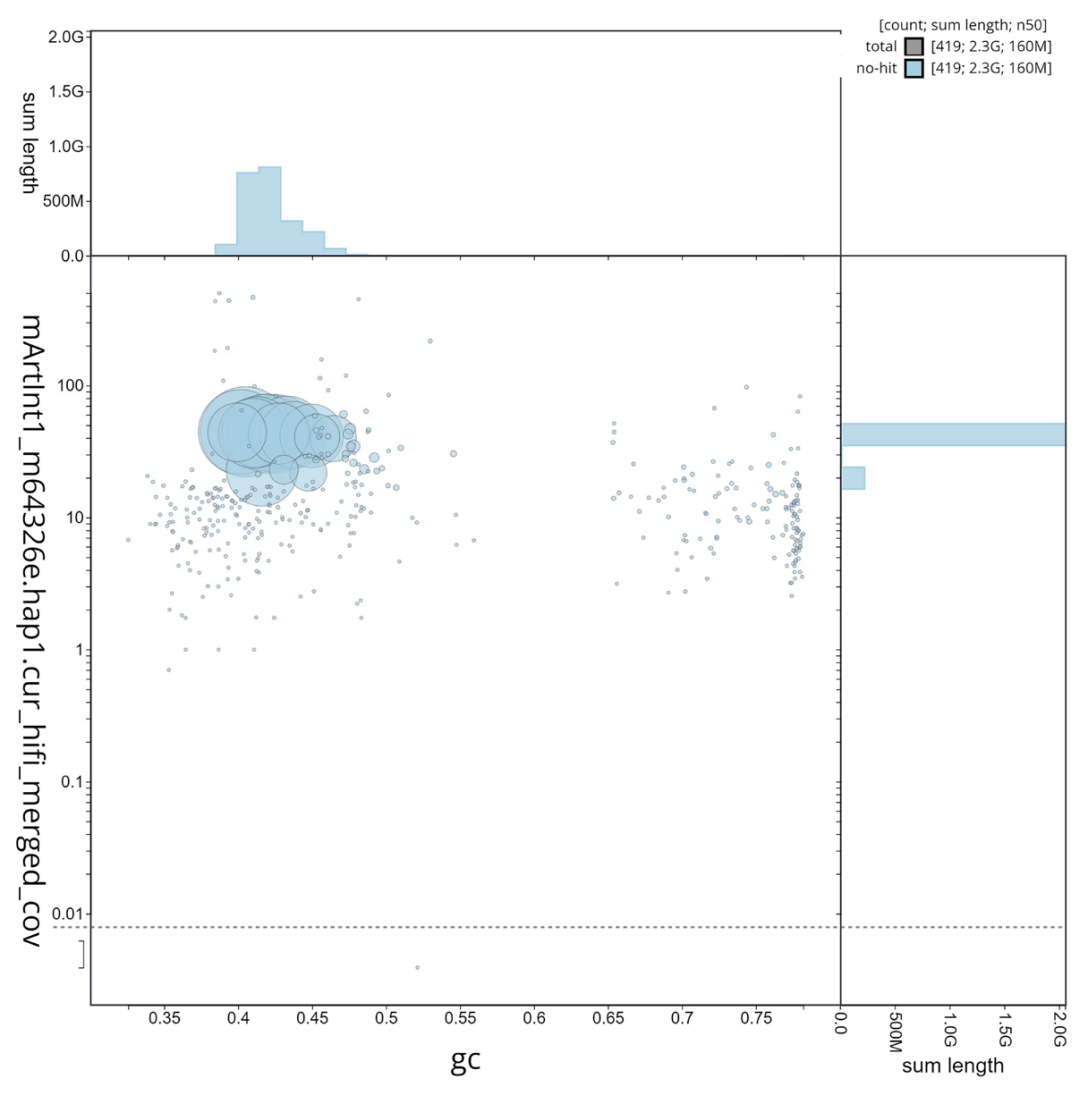
GC coverage plot generated for the *Artibeus intermedius* assembly using blobtoolkit. Individual chromosomes and scaffolds are represented by each circle. The circles are sized in proportion to chromosome/scaffold length. Histograms show the sum length of chromosome/scaffold size along each axis. Color of circles indicate taxonomic hits of each Phylum represented in the assembly. Done in collaboration with Excelra through Paratus Sciences.

**Figure 6.**
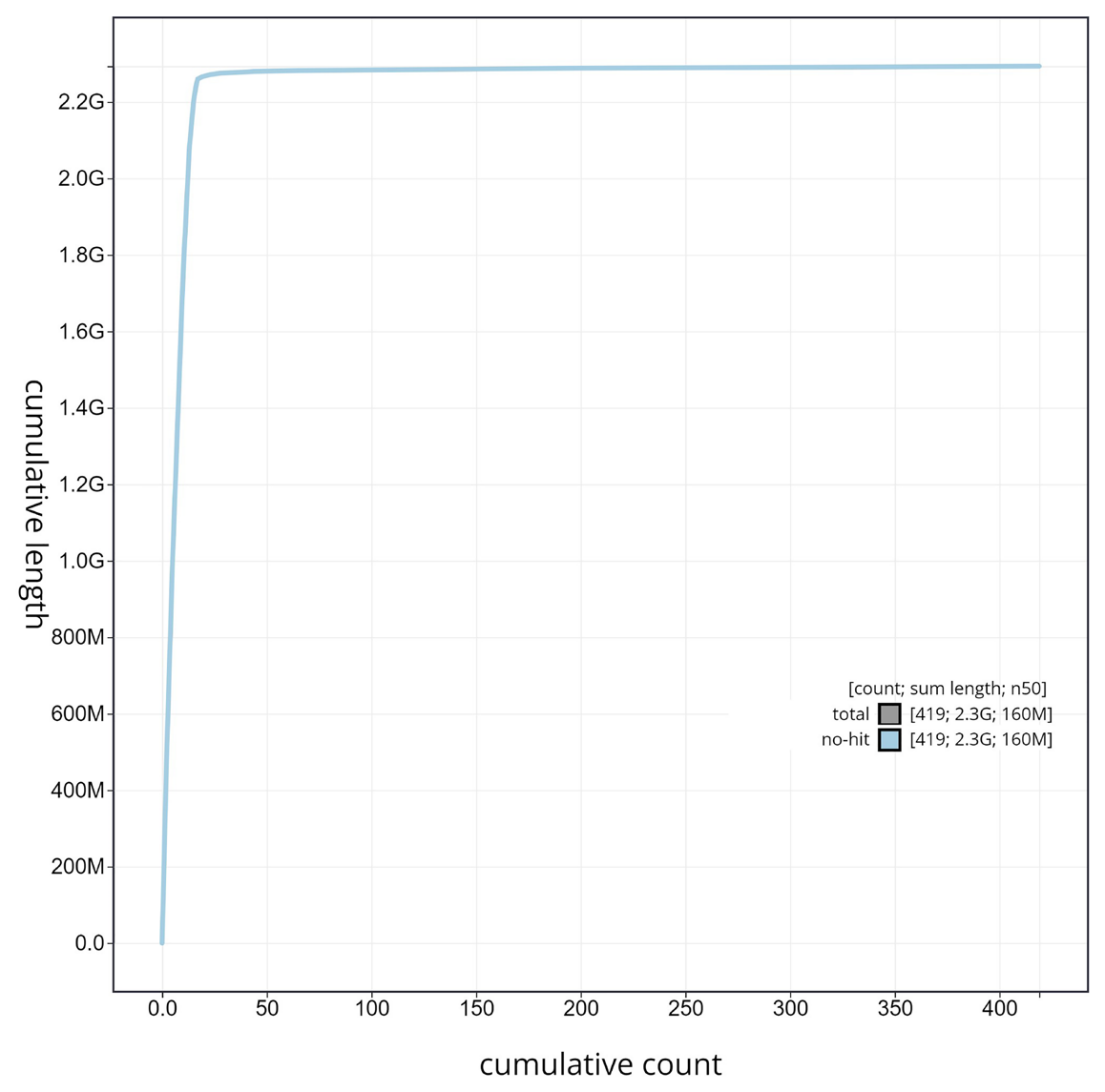
Cumulative sequence plot generated for the *Artibeus intermedius* assembly using blobtoolkit. The grey line shows the cumulative length for all chromosomes/scaffolds in the assembly. Colored lines represent Phylum represented in the assembly. Done in collaboration with Excelra through Paratus Sciences.

**Table 3.** Software tools used.

Software tool	Version	Source
bamUtil	1.0.15	https://genome.sph.umich.edu/wiki/BamUtil:_bam2FastQ
MultiQC	1.13	https://github.com/ewels/MultiQC
Genomescope	2.0	https://github.com/tbenavi1/genomescope2.0
hifiasm	0.19.3	https://github.com/chhylp123/hifiasm
purge_dups	1.2.6	https://github.com/dfguan/purge_dups
BUSCO	5.3.2	https://busco.ezlab.org/
Merqury	1.3	https://github.com/marbl/merqury
Assembly-stats	17.02	https://github.com/rjchallis/assembly-stats
Arima-HiC Mapping Pipeline	-	https://github.com/ArimaGenomics/mapping_pipeline
YaHS	1.1	https://github.com/c-zhou/yahs
HiGlass	1.11.7	https://github.com/higlass/higlass
samtools	1.9	https://www.htslib.org/
PretextView	-	https://github.com/sanger-tol/PretextView/tree/master
BUSCO	5.7.0	https://busco.ezlab.org/

## Ethics and consent

Capture and sampling were conducted under Belize Forest Department Permit FD/WL/1/21(16) and Belize Institute of Archaeology Permit IA/S/5/6/21(01), and samples were exported under Belize Forest Department permit FD/WL/7/22(08). All work was conducted with approval by the AMNH Institutional Animal Care and Use Committee (AMNHIACUC-20190129, date of approval 29 ^th^ January 2019. date of approval 29 ^th^ January 2019.

## Data Availability

The *Artibeus intermedius* genome sequencing initiative is part of the Bat1K genome sequencing project. The genome assembly is released openly for reuse. Please email info@bbf.org for more information. European Nucleotide Archive (ENA): Genome assembly of *Artibeus intermedius* as part of the Bat1K genome sequencing project. Accession number GCA_038363145.1; https://www.ebi.ac.uk/ena/browser/view/GCA_038363145.1 ( BAT1K, 2024a). European Nucleotide Archive (ENA): Genome assembly of *Artibeus intermedius*, haplotype 2. Accession number GCA_038363225.1; https://www.ebi.ac.uk/ena/browser/view/GCA_038363225.1 ( BAT1K, 2024b). NCBI: Genome assembly of *Artibeus intermedius*, haplotype 1 (mArtInt1.hap1). Accession number GCA_038363145.1; https://www.ncbi.nlm.nih.gov/datasets/genome/GCA_038363145.1/ ( BAT1K, 2024a). NCBI: Genome assembly of *Artibeus intermedius*, haplotype 2 (mArtInt1.hap2). Accession number GCA_038363225.1; https://www.ncbi.nlm.nih.gov/datasets/genome/GCA_038363225.1/ ( BAT1K, 2024a). Data accession identifiers are reported in Table 2. The ARRIVE checklist for this study has been deposited in Zenodo and is publicly accessible. Repository name: Zenodo; Checklist for "The genome sequence of *Artibeus intermedius* (Chiroptera, Phyllostomidae, Stenodermatinae; J. A. Allen, 1897), DOI: 10.5281/zenodo.14909648; ( BAT1K, 2025) License: Creative Commons Attribution 4.0 International
