# Supplementary material for: The genome sequence of Artibeus intermedius (Chiroptera, Phyllostomidae, Stenodermatinae; J. A. Allen, 1897)
Source: Wellcome Open Res. 2025 Jun 9;10:314. [Version 1] doi: 10.12688/wellcomeopenres.23865.1 (PMC12648033; doi:10.12688/wellcomeopenres.23865.1)
Supplement: Peer review history

# Reviewer report: Reviewer response for version 1

**DOI:** 10.21956/wellcomeopenres.26330.r139207

**Published:** 2025-12-08

**Authors:** M. Alejandra Camacho

**Affiliations:** 1 Pontificia Universidad Catolica del Ecuador, Quito, Pichincha, Ecuador

This report presents a high-quality, chromosome-scale genome assembly for *Artibeus intermedius*, generated using PacBio HiFi long reads and Hi-C data. This genome represents an important contribution to the Bat1K project and provides a valuable resource for future studies on phyllostomid evolution. The manuscript is well prepared and clearly written. The methods follow current standards for vertebrate genome assembly, and the authors provide metadata, accession numbers, and sample documentation.

 I commend the authors for the quality of the assembly and for their adherence to Bat1K protocols. I identified a few points that should be addressed to improve clarity, consistency, and reproducibility. I added some comments in a reviewed ms. These are mostly minor but necessary before indexing.

 1. Clarification on contamination screening procedures (Methods). The text does not mention whether any scaffolds were identified as contamination or removed.

 2. The report states that haplotype 1 was selected as the reference due to “higher quality metrics,” but the specific metrics are not reported. Please include the relevant comparisons, and indicate whether haplotype 2 presented structural issues.

 3. Table 1 (page 5) lists the chromosome number of A. intermedius as “2n = [15].” As Reviewer 1 noted, this likely refers to the haploid number (n = 15). Please revise the notation and consider adding a brief statement referencing available cytogenetic data.

 4. Check BUSCO versions consistency.

 5. The manuscript does not explicitly state whether any annotation was generated. If the assembly is intentionally released without annotation please add a sentence confirming this.

 6. The Hi-C map lacks chromosome number labeling, and only 16 pseudochromosomes are clearly visible. Adding labeling or clarifying the map scaling would improve readability.

 7. Minor typographical issues. Please revise:

 Pages 3 and 4: A. literatus. Correct: A. lituratus

 Page 4. Cercropia. Correct: Cecropia

 Page 4: Stenocerus. Correct: Stenocereus

Are sufficient details of methods and materials provided to allow replication by others?

Yes

Is the rationale for creating the dataset(s) clearly described?

Yes

Are the datasets clearly presented in a useable and accessible format?

Yes

Are the protocols appropriate and is the work technically sound?

Yes

Reviewer Expertise:

Neotropical Bats taxomomy and evolution.

I confirm that I have read this submission and believe that I have an appropriate level of expertise to confirm that it is of an acceptable scientific standard, however I have significant reservations, as outlined above.

# Reviewer report: Reviewer response for version 1

**DOI:** 10.21956/wellcomeopenres.26330.r139209

**Published:** 2025-11-25

**Authors:** Xiuguang Mao

**Affiliations:** 1 East China Normal University, Shanghai, China

Simmons et al. present a high-quality chromosome-scale assembly for *Artibeus intermedius* using Pacific Biosciences Hi-Fi long reads and Hi-C reads. This genomic resource offers valuable insights into molecular divergence and dietary adaptation in Phyllostomidae. For the current version of the manuscript, I have three minor comments:

 1）In the text "...generate contigs of each haplotype 1 (mArtInt1.hap1) and haplotype 2 (mArtInt1.hap1)", the reference to haplotype 2 should be corrected to "mArtInt1.hap2".

 2）The sentences describing BUSCO scores below Table 2 should be relocated to Figure 4. Additionally, please specify which BUSCO database was used—mammalia_odb10 or laurasiatheria_odb10—and ensure consistency in both the genome report and Figure 4.

 3）The chromosome number for *Artibeus intermedius* is stated as "2n=[15]", but it should be revised to "n=[15]" to indicate the haploid number.

Are sufficient details of methods and materials provided to allow replication by others?

Yes

Is the rationale for creating the dataset(s) clearly described?

Yes

Are the datasets clearly presented in a useable and accessible format?

Yes

Are the protocols appropriate and is the work technically sound?

Yes

Reviewer Expertise:

Comparative genomics, genome assembly, evolutionary biology, speciation, bats

I confirm that I have read this submission and believe that I have an appropriate level of expertise to confirm that it is of an acceptable scientific standard.

# Reviewer report: Reviewer response for version 1

**DOI:** 10.21956/wellcomeopenres.26330.r125586

**Published:** 2025-06-23

**Authors:** Tinglei Jiang, Yang Geng

**Affiliations:** 1 Northeast Normal University, Changchun, Changchun, China

The Bat1K project has catalyzed advancements in understanding bat adaptive evolution. As part of this initiative, this research presents the high-quality genome assembly of *Artibeus intermedius*, a frugivorous bat species. Utilizing PacBio HiFi long-read sequencing and Hi-C scaffolding, this research generated a chromosomal-level assembly comprising 17 pseudochromosomes, including the sex chromosomes (X and Y1/Y2). The final assembly spans 2.3 Gb with a scaffold N50 of 160.4 Mb and exhibits high completeness. This resource resolves taxonomic ambiguities surrounding *A. intermedius *and provides a foundation for investigating its biology, ecology, and interactions with viral pathogens.

 However, minor revisions are required to address before indexing. These adjustments will strengthen the manuscript’s clarity, reproducibility and robustness.

1. The authors describe that the genome assembly of *​* **​** *Artibeus intermedius*​ *​ *contains 17 pseudochromosomes. It would be beneficial to incorporate karyotypic data for ​ **​**A. intermedius​​. For example, refer to Yonenaga et al. (1969) (Ref 1)
2. The Hi-C contact map lacks chromosome number labeling, reducing readability. Only 16 pseudochromosomes are clearly visible.
3. The authors mention using Merqury to assess assembly quality but fail to include corresponding results in the text, figures, or tables.
4. Table 3 lists two BUSCO software versions, and conflicting citations of BUSCO in the "Genome Sequence Report" and Methods sections. Please reconcile discrepancies across all sections.
5. The text states 96.5% BUSCO completeness of A. intermedius genome assembly **,** whereas Figure 4 reports 97.6%. Please check this discrepancy.

Are sufficient details of methods and materials provided to allow replication by others?

Yes

Is the rationale for creating the dataset(s) clearly described?

Yes

Are the datasets clearly presented in a useable and accessible format?

Yes

Are the protocols appropriate and is the work technically sound?

Yes

Reviewer Expertise:

Behavioral Ecology; Comparative genomics; Evolution biology

We confirm that we have read this submission and believe that we have an appropriate level of expertise to confirm that it is of an acceptable scientific standard.

## References

[1] : Karyotypes of Seven Species of Brazilian Bats. *Caryologia* .1969;22(1) : 10.1080/00087114.1969.10796325 63-79 10.1080/00087114.1969.10796325
